# Eye movement analysis of children’s attention for midline diastema

**DOI:** 10.1038/s41598-022-11174-z

**Published:** 2022-05-06

**Authors:** Vanessa Y. Cho, Janet H. Hsiao, Antoni B. Chan, Hien C. Ngo, Nigel M. King, Robert P. Anthonappa

**Affiliations:** 1 UWA Dental School, The University of Western Australia, 17 Monash Avenue, Nedlands, WA 6009 Australia; 2 Department of Psychology, University of Hong Kong, Pok Fu Lam, Hong Kong SAR; 3 Department of Computer Science, City University of Hong Kong, Kowloon Tong, Hong Kong SAR

**Keywords:** Dental diseases, Oral diseases

## Abstract

No previous studies have investigated eye-movement patterns to show children’s information processing while viewing clinical images. Therefore, this study aimed to explore children and their educators’ perception of a midline diastema by applying eye-movement analysis using the hidden Markov models (EMHMM). A total of 155 children between 2.5 and 5.5 years of age and their educators (n = 34) viewed pictures with and without a midline diastema while Tobii Pro Nano eye-tracker followed their eye movements. Fixation data were analysed using data-driven, and fixed regions of interest (ROIs) approaches with EMHMM. Two different eye-movement patterns were identified: explorative pattern (76%), where the children’s ROIs were predominantly around the nose and mouth, and focused pattern (26%), where children’s ROIs were precise, locating on the teeth with and without a diastema, and fixations transited among the ROIs with similar frequencies. Females had a significantly higher eye-movement preference for without diastema image than males. Comparisons between the different age groups showed a statistically significant difference for overall entropies. The 3.6–4.5y age groups exhibited higher entropies, indicating lower eye-movement consistency. In addition, children and their educators exhibited two specific eye-movement patterns. Children in the explorative pattern saw the midline diastema more often while their educators focussed on the image without diastema. Thus, EMHMMs are valuable in analysing eye-movement patterns in children and adults.

## Introduction

Diastema refers to a gap or space between two or more consecutive teeth. A midline diastema refers to a space between the maxillary central incisors. Approximately 40% of preschool children exhibit generalised spacing between the primary maxillary incisors^1^. However, causes such as abnormal anatomy of the maxillary labial frenum, midline bony clefts, non-nutritive sucking habits, physical impediments and dental anomalies may lead to a diastema^2^. Nevertheless, little is known about children’s perception of a midline diastema. One cannot prove objectively if children even notice such dental variations to this extent.

Understanding children’s expression may be limited by vocabulary, comprehension of words, relatively little world experience, and shorter attention span^3^. What preschool-age children notice may differ from adults, or what we assume children may notice may vary from what they see. This adaptive mechanism of distributing attention can be advantageous to adults, who often employ selective attention to focus on essential data^4^. Nevertheless, studies involving children often rely on an adult interpretation, leading to the proxy effect leading to under or over-reporting events and the saliency principle effect where proxy persons recall more accurately or are relevant in their reporting^5^.

As a well-validated research tool, eye-tracking technology provides accurate, objective, and real-time measures of visual fixation, gaze pattern, and pupil response to understand children’s perception^6,7^. Recently, a novel eye-movement analysis with hidden Markov models (EMHMM) has been developed (Hidden Markov model is a time-series statistical model in machine learning. EMHMM Matlab toolbox is available at http://visal.cs.cityu.edu.hk/research/emhmm/)^8^. This method incorporates individual differences in spatial (eye fixation locations) and temporal dimensions (the order of eye fixation locations) of eye movements to provide a quantitative measure of individual differences in the eye movement pattern. Furthermore, combining EMHMM with the data mining technique, co-clustering facilitates identifying participant groups with consistent eye-movement patterns while viewing clinical images with varying layouts^9^. Thus, this innovative approach explores individual differences in eye movement patterns and their associations with the participant’s cognitive measures^10^.

To date, no studies have addressed what young children notice when they see individuals with or without a midline diastema. Also, it is unclear at what age children notice, if they do, a midline diastema. Hence, objective data of where children fixate when looking at images with or without diastema will provide better information regarding children’s perception of such dental variations. Therefore, this study aimed to explore individual differences among preschool children and their educator’s eye movement patterns and visual attention to images with and without a midline diastema via EMHMM. We hypothesised that a picture with a midline diastema would hold or capture visual attention more effectively than an image without a midline diastema.

## Material and methods

The University of Western Australia Human Research Ethics Committee (2019/RA/4/1/9331) granted the following Ethics approval. This study followed the STROBE guidelines. The sample size was calculated using G*Power (Version 1.4.1) based on Tanaka and co-workers’ results^10^ for a significance level of alpha = 0.05 and 90% power. This estimation indicated 103 children were required.

We recruited children and educators from 13 different childcare centres in metropolitan Perth, Western Australia. Five centres within the central business district and three, five- and ten-kilometre radii were randomly selected and subsequently contacted. Participants received information outlining the project details, and their parents or legal guardians provided written informed consent before study commencement. For comparisons, educators from the same childcare centres participated following a consent process.

Before viewing the pictures, all children were presented with two activities [(i) complete the pattern and (ii) join the dots in a line (Appendix Fig. 1.) to achieve in their own time. We used these activities to group children in ways other than age to test their ability to follow instructions and manual dexterity. This strategy facilitated the data to be analysed based on gender (male, female), age (2.6–3.5y, 3.6–4.5y, and 4.6–5.5y) and the activity (complete or incomplete). A total of 155 children born in Australia participated in this study. However, no significant differences were evident for all demographic variables, as shown in Table 1 and Appendix Table 1. Similarly, 34 educators participated, and they did not complete any activities before viewing the images. All children were born in Australia from different cultural backgrounds, and there was no statistical significance between the groups.

**Table 1:** Study participants details based on gender, age and activity completion.

Gender	Activity	Age (years)			Total
		2.6–3.5	3.6–4.5	4.6–5.5	
Male	Incomplete	21	9	0	30
n = 83	Complete	9	21	23	53
Female	Incomplete	16	3	0	19
n = 72	Complete	8	19	26	53
Total		54	52	49	155

**Figure 1:**
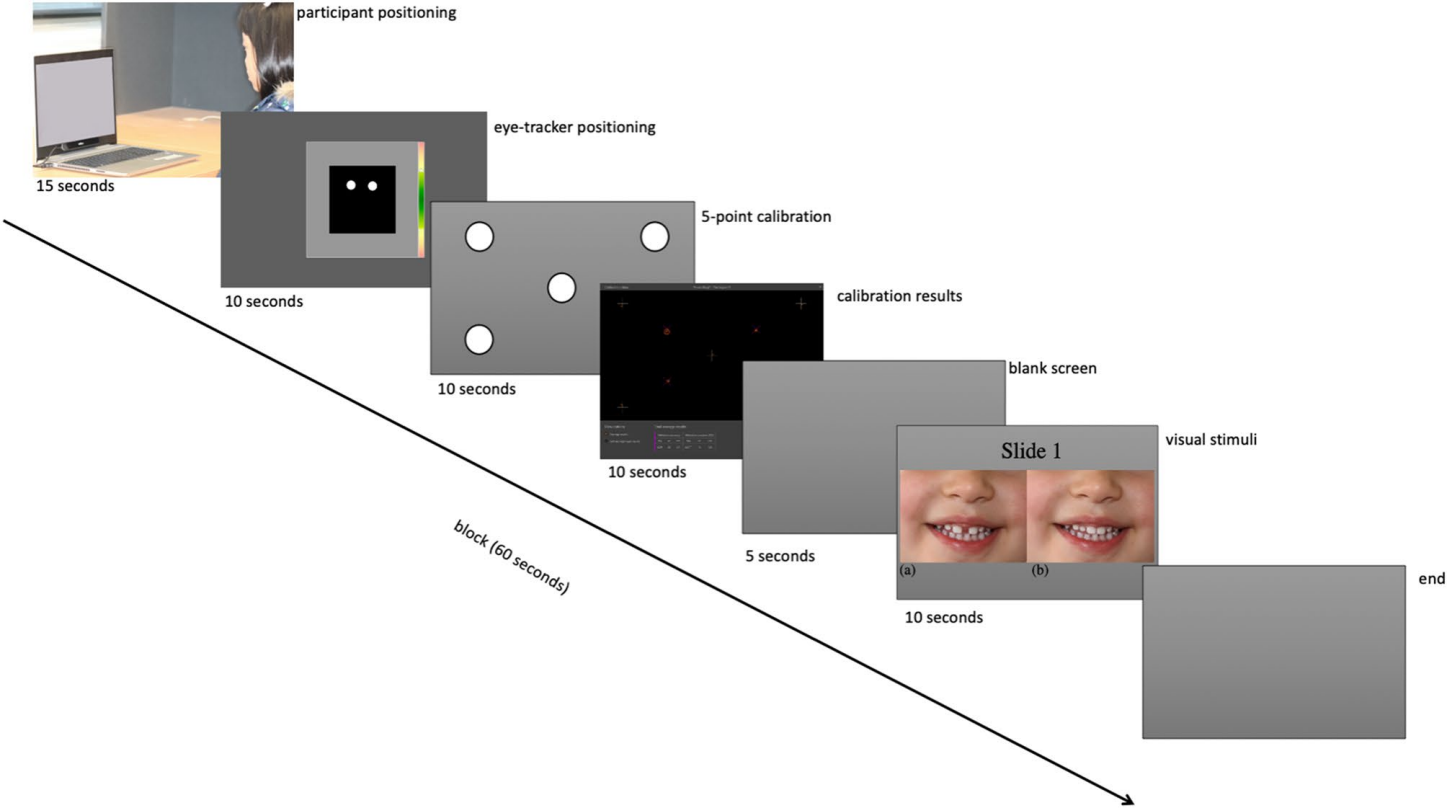
Timeline of procedure showing Slide 1: photographs (**a**) with a diastema and (**b**) without diastema used to assess the participant’s eye movement patterns.

### Setting and equipment

The Tobii Studio software (Tobii, Danderyd, Sweden) was installed onto a Hewlett Packard ProBook 640 G2 laptop computer (Hewlett Packard, Boeblingen, Germany) and set up in the childcare centres. A Tobii Pro Nano screen-based eye-tracking camera (Tobii, Danderyd, Sweden), recording at 60 frames per second, was connected to the laptop computer placed on a desk. The participants independently viewed the images in a quiet room seated on a stable chair approximately 30–50 cm away from the laptop. All participants viewed the same pictures, as shown in Fig. 1. i.e. two photographs, one with and another without a midline diastema.

Participants were positioned in the middle of the camera’s field of view, and the chair used was not height-adjustable to prevent untoward movements. We adjusted the screen and camera to capture the participants’ eyes movements. The investigation started with a five-point eye-tracker calibration exercise, using an animation video of a duck that made an alerting sound to gain attention and moved across the screen, taking approximately 10 s. This task allowed for standardisation among the participants and also calibrated the participants’ eyes to the eye-tracker. Subsequently, each participant viewed the image (Fig. 1) and did not perform other tasks until the screen went blank, indicating the end. Also, the instructions were straightforward to avoid differing interpretations of the task at hand. Later, we exported the fixation points for each participant into an excel spreadsheet.

### Data analysis

The eye-fixation data was analysed using EMHMM, which incorporated individual differences in spatial (eye-fixation locations) and temporal dimensions (the order of eye-fixation sites). We used data-driven and fixed ROI methods to assess participants’ eye movement patterns. We assumed each ROI to follow a 2D Gaussian distribution. The data-driven approach generated ROIs that best fit the distributions of eye fixations of the participants in the present study. Conversely, the fixed ROI method required the investigators to define the ROIs. A two-tailed t-test was used to compare means for normally distributed and skewed data with the P values set at 0.05 for statistical significance. Also, we measured the overall entropies to determine the eye movement consistencies. T-test was computed to compare eye movement pattern and consistency measures between gender and activity groups, while ANOVA was used for age groups.

Using EMHMM, each participant’s eye movements were summarised using a hidden Markov model (HMM), with its hidden states corresponding to the viewed regions of interest (ROIs). A hidden state sequence generated a sequence of the viewed ROIs under a Markov process. Person-specific ROIs were identified based on the eye fixation distributions. The transitions among the ROIs were summarised into a transition matrix showing the probability of eye gaze moving from a previously viewed ROI to the current ROI. Subsequently, individual HMMs were clustered into groups according to the similarities of their ROIs and ROI sequences to discover representative eye movement patterns among the participants.

Following previous studies^8,11–16^, we clustered individual HMMs into two representative patterns: Pattern 1 and Pattern 2. The similarity of a participant’s eye movement data to a suggestive pattern could be quantified using the log-likelihood of the participant’s eye movement data being generated by the representative model. We then assessed each participant’s eye movement pattern using a 1–2 scale, defined as (L1 − L2)/(|L1| +|L2|), where L1 and L2 stand for the log-likelihoods of the participant’s eye movement data being generated by Pattern 1 and Pattern 2 respectively^13–16^. Finally, eye movement consistency was assessed using the HMM’s overall entropy, where entropy was a measure of regularity or predictability of eye movements^17^. For example, a higher entropy indicated more randomness or variability within an individual’s eye movements^18^. Raw data were analysed using EMHMM11 and GraphPad Instat (California, USA).

We cleaned the pupil diameter data to eliminate outlier values corresponding to blinks or any large movements in the head position or if the participant did not look at the image for three or more consecutive 300 ms intervals (rendering the baseline pupil diameter invalid). Next, mean pupil diameter was calculated for interval durations of 300 ms across the 10 s presentation, with time-locked to the onset of the image. Next, we used the following calculation to compute the pupillary response: baseline pupil diameter (taken at the first 300 ms) during the start of the presentation interval—maximum pupil diameter during the presentation. Finally, the difference value was divided by the baseline pupil diameter to determine a percentage increase in pupil diameter.

## Results

### Children data-driven method

**Figure 2:**
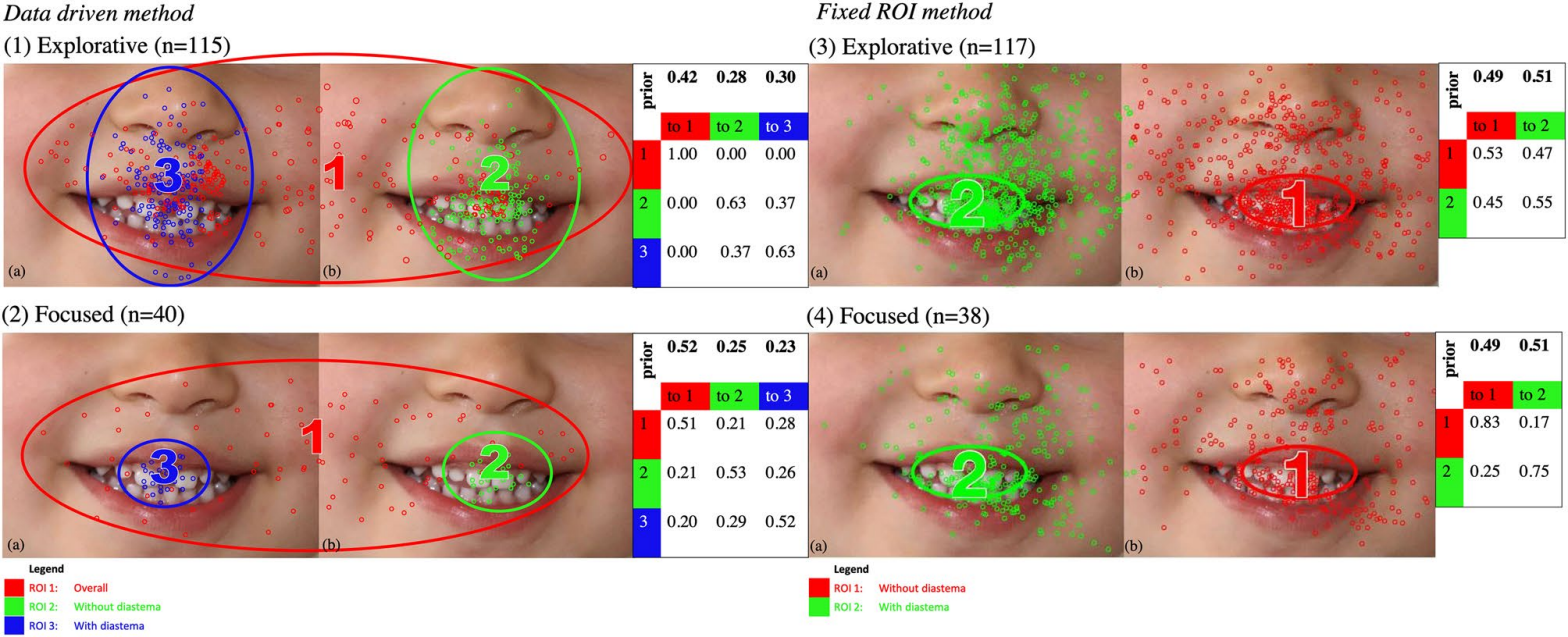
The two different eye movement patterns were identified in children using the data-driven and fixed ROI methods. Children data-driven method: (1) explorative pattern, where children’s ROIs were predominantly around the nose and mouth, and (2) focused pattern, where children’s ROIs were mainly on the teeth, i.e. images with and without a diastema. Ellipses show two standard deviation contours from the mean of the ROIs (2D Gaussian distributions). Small circles in different colours show the assignment of raw fixations to different ROIs. The table shows transition probabilities among the ROIs. Priors show the probabilities that a fixation sequence starts from an ROI. For example, children in the explorative pattern showed a 42% chance of the first fixation in ROI1, 28% chance in ROI2, and 30% in ROI3. After that, they remained in the same ROI (ROI1 = 100%, ROI2 = 63%, ROI3 = 63%) or switched ROI (ROI2 to ROI3 = 37% and ROI3 to ROI2 = 37%). Children fixed ROI method: (3) explorative pattern, where children tended to switch between the two images more often and (4) focused pattern, where children looked at the same image for a while before switching to the other image. The ROIs are defined as elliptical areas in the mouth region and represented as ROI1: without diastema and ROI2: with diastema. Note: small circles show raw fixation locations, and the colour of the small circles indicate ROI assignments. Priors indicate the probability that a fixation sequence starts from the corresponding ROI. The transition matrix suggests the likelihood of eye gaze transits between the ROIs. For example, in the explorative pattern (3) group, children showed a 49% chance of their first fixation in ROI1 or 51% chance in ROI2. After that, they remained in the same ROI (ROI1 = 53%, ROI2 = 55%) or switched ROI (ROI1 to ROI2 = 47% and ROI2 to ROI1 = 45%).

Two different eye movement patterns were identified in children using this method through clustering: Pattern 1, the explorative pattern (76%), where the children’s ROIs were predominantly around the nose and mouth, and Pattern 2, the focused pattern (26%), in which children’s ROIs were precise, locating on the teeth with and without a diastema, and fixations transited among the ROIs with similar frequencies (Fig. 2). A two-tailed t-test revealed a statistically significant difference between these two eye movement patterns. i.e., data from participants using the explorative pattern had a significantly higher log-likelihood to generate the explorative than the focused HMM and vice versa for data from those using the focused pattern. Here we referred to the 1–2 scale as E–F (Explorative-Focused) scale and used it to quantify participants’ eye movement patterns and the contrast between the explorative and focused patterns.

In the explorative pattern (n = 115), ROI1 included both images with and without diastema, ROI2 consisted of the circumoral region (nose and mouth) without diastema, and ROI3 included the circumoral area with a diastema (Fig. 2 1a and b). Children adopting this pattern had a 42% probability of viewing the image with the first fixation in ROI1, 28% in ROI2, and 30% in ROI3. Furthermore, once they began from ROI1, they stayed in ROI1 for the entire duration, i.e. looking broadly at both images. Conversely, if they started in ROI2 or ROI3, they either remained in the same ROI (63%) or switched to the other ROI in the subsequent fixation (37%); they consistently transited among the ROI2 and ROI3.

Similarly, ROI1 covered both pictures with and without diastema for the focused pattern (n = 40), ROI2 teeth with diastema and ROI3 without diastema (see Fig. 2 2a and b). Children adopting this pattern had a 52% probability of starting in ROI1, 25% in ROI2, and 23% in ROI3. However, once they began their fixation in ROI1, only 51% remained in ROI1, while 21% moved to ROI2 and 28% to ROI3. Similarly, if they started from ROI2, 53% of the time, they continued staying in ROI2, while 21% moved to ROI1 and 26% to ROI3. Finally, if they started from ROI3, 52% of the time, they continued staying in ROI3, while 20% moved to ROI1 and 29% to ROI2. Thus, children adopting this pattern transited among the 3 ROIs.

Overall entropies of the two eye movement pattern groups were significantly different. A Pearson R correlation test revealed no statistical significance between overall entropy and the E–F scale (Pearson r = 0.23, 95% CI =  − 0.38–0.12). Comparisons between the different age groups showed a statistically significant difference [one way ANOVA] for overall entropies only between 3.6 and 4.5y and 4.6–5.5y age groups with the 3.6–4.5y age groups higher entropies (mean = 11.89, MD = 0.85, SEM = 0.27) than 4.6y–5.5y are groups, [F(2,153) = 4.94, *p* = 0.01], indicating lower eye movement consistency. Furthermore, no significant differences were evident for entropies between males and females for all the age groups.

### Children fixed ROI method

**Figure 3:**
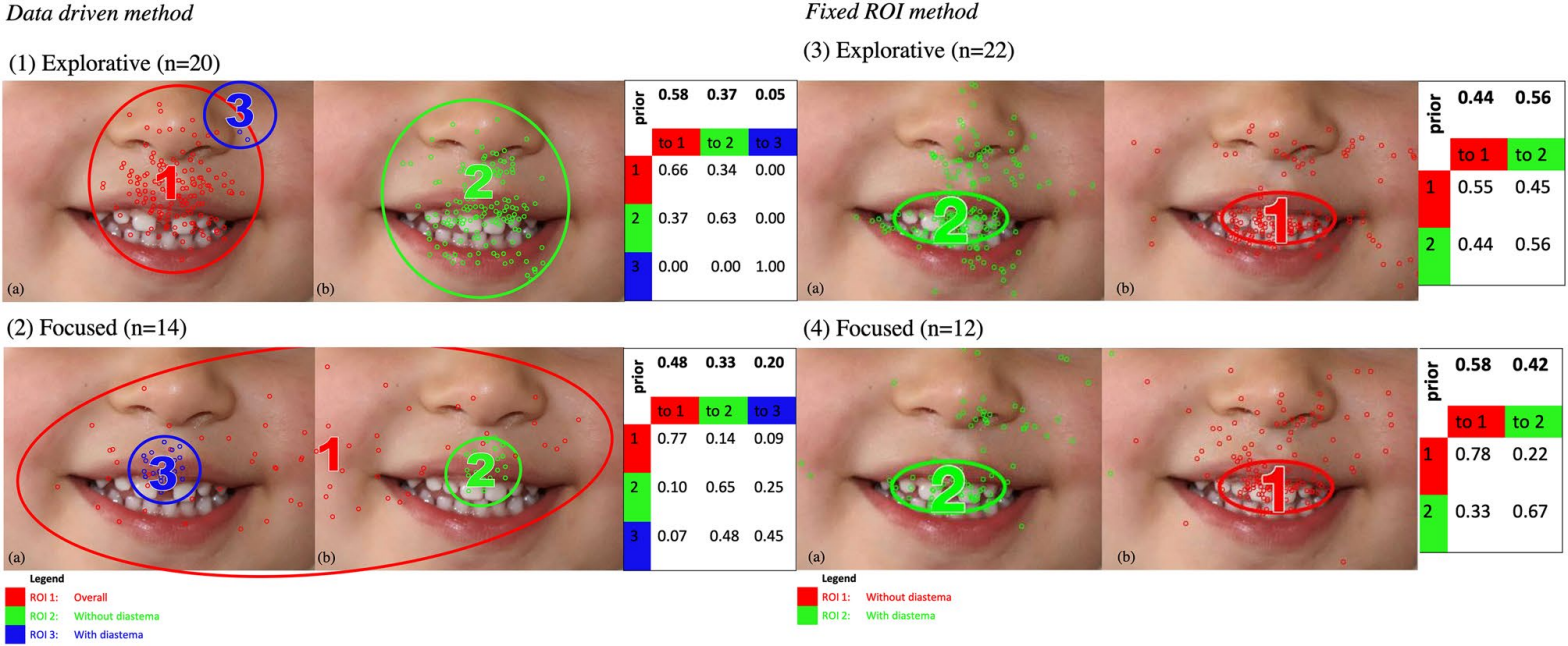
The educators exhibited two different eye movement patterns: (1) explorative pattern, where the ROIs were predominantly around the nose and mouth, and (2) focused pattern, where the ROIs were mainly on the teeth. Fixed ROI method: illustrates the two fixed teeth-focused ROIs and the two different eye movement patterns identified in educators using the fixed ROI method. (3) Explorative pattern, where participants tended to switch between the two images more often. (4) Focused pattern, where participants tended to view the same image longer before switching to the other image. The ROIs are defined as elliptical areas in the mouth region and represented as ROI1: without diastema and ROI2: with diastema. Ellipses show two standard deviation contours from the mean of the ROIs (2D Gaussian distributions). Small circles in different colours show the assignment of raw fixations to different ROIs. The table shows transition probabilities among the ROIs. Priors show the probabilities that a fixation sequence starts from an ROI. For example, in the fixed ROI explorative pattern (3) group, educators had a 44% chance of starting fixations in ROI1 and a 56% chance of starting in ROI 2. After that, they remained in the same ROI (ROI1 = 55%, ROI2 = 56%) or switched ROI (ROI1 to ROI2 = 45% and ROI2 to ROI1 = 44%).

**Figure 4:**
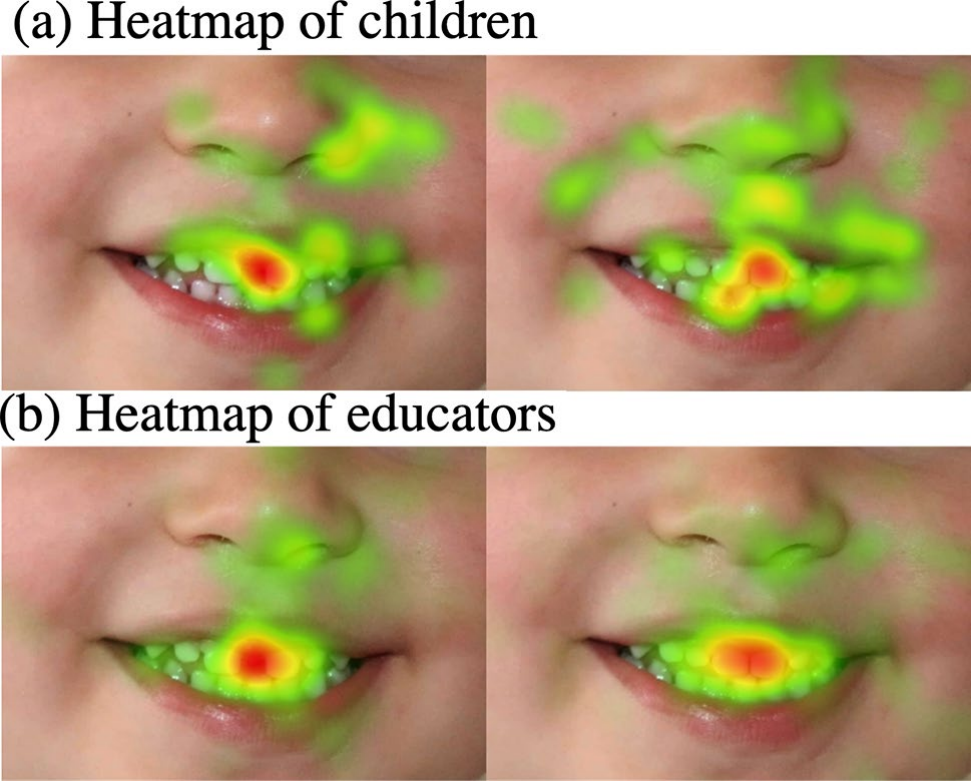
Heatmaps illustrating the areas of fixation for (**a**) children (**b**) educators when they viewed images with and without diastema. Note area in red indicates the maximum focus, which is predominantly on teeth for both groups.

We focused our analysis on the gaze transition behaviour between the two images in the fixed ROI method. The two ROIs defined were teeth-focused, with an ellipse representing the two standard deviation contour from the mean of the 2D Gaussian distribution (Fig. 2- 3 and 4). The ROI1 ellipse outlined the teeth without diastema and ROI2 teeth with diastema. EMHMM classified all fixations into ROI1 and ROI2 according to their likelihoods of being generated from the ROI1 or ROI2 2D Gaussian distributions. Since ROIs were fixed, the difference between the two patterns was mainly in the transition matrix, showing how children’s eye gaze transited between the two images (Fig. 2- 3 and 4). Again, clustering results showed that the two different eye movement pattern groups were evident. Pattern 1 as an explorative pattern and Pattern 2 as a focused pattern since Pattern 1 involved higher probabilities to transit between the two images than Pattern 2 (Fig. 2- 3 and 4). In both patterns, participants showed a 51% probability of starting on the image without diastema (ROI1) and a 49% probability of beginning on the image with diastema (ROI2), see Fig. 2- 3 and 4. A two-tailed t-test confirmed statistical significance between the two eye movement patterns (*P* < 0.05).

In the explorative pattern (n = 117), children switched between diastema or no diastema images around 50% of the time. Conversely, in children exhibiting a focused eye movement pattern (n = 38), if they started in the no diastema image (ROI1), they stayed looking at the no diastema image with 83% probability. In contrast, with 17% probability, they switched to the diastema image (ROI2). If they started in the diastema image (ROI2), they moved to the no diastema image (ROI1) 25% of the time, whereas 75% stayed fixating on the diastema image (ROI2).

Overall entropy between the two eye movement pattern groups was not significantly different [*t*(153) = 0.21, *p* = 0.84]. The overall entropies were also not quite different between gender [*t*(153) = 1.28, *p* = 0.20], activity [*t*(153) = 1.91, *p* = 0.06], or age groups [F(2,153) = 0.6, *p* = 0.82]). Similarly, the E–F scale was not statistically significant difference between gender [*t*(153) = 0.90, *p* = 0.37], activity [*t*(153) = 0.16, *p* = 0.87], or age groups [F(2,153) = 0.49, *p* = 0.61]. T-test to compare gaze preference of no diastema between explorative and focused groups revealed that children with an explorative eye movement pattern (mean = 0.47 in explorative, mean = 0.56 in focused groups, SEM = 0.04) had a lower gaze preference for the no diastema image (ROI1; quantified by the percentage of fixations classified as belonging to ROI1/the no diastema image) than those in the focused pattern group [*t*(153) = 2.69, *p* = 0.01]. The gaze preference was then compared to a 50% and one-sample t-test. For the explorative pattern group, the mean was 0.47 [*t*(116) = 2.24, *p* = 0.02], therefore, children in this group had a lower gaze preference for the no diastema image than the diastema image (or, in other words, they looked at the diastema image more often than the no diastema image). The mean was 0.56 [*t*(37) = 1.41, *p* = 0.16] in the focused pattern group, indicating that children in this group did not have a significant bias to either image with or without diastema. Females (mean = 0.53) had a statistically significant higher gaze preference than males (mean = 0.46) for ROI1, i.e. without diastema image [*t*(153) = 2.37, *p* = 0.02]. There was no statistically significant difference in gaze preference for no diastema image (ROI1) for the different ages [F(2,153) = 0.06, *p* = 0.94] or activity groups [*t*(153) = 1.37, *p* = 0.17].

The increase in mean pupil diameter is illustrated in Table 2, where the percentage increase in pupil diameter had no significant difference between explorative and focused eye movement pattern groups [*t*(67.19) = 0.09, *p* = 0.92]. There was no significance difference between between eye movement pattern groups among females [*t*(18.52) = 0.90, *p* = 0.65] or males [*t*(40.75) = 0.28, *p* = 0.78].

**Table 2:** Shows the children and their educators mean pupil diameter increase (%) when they viewed images of a midline diastema and without diastema.

	Explorative pattern	Focused pattern	*p*	t	df	MD ± SEM	95% CI
Children	11.48	11.28	0.92	0.09	67.19	0.19 ± 1.9	− 3.68 to 4.06
Male	10.90	10.18	0.78	0.28	40.75	0.72 ± 2.54	− 4.40 to 5.38
Female	12.47	9.86	0.65	0.90	18.52	2.61 ± 2.91	− 3.49 to 8.72
Educators	15.76	11.65	0.39	0.88	28.46	4.11 ± 4.67	− 5.44 to 13.66

T-test with significance at *p* < 0.05.

*p* = *p*-value, *t* = t-value, *df* = Degrees of freedom, *MD* = Mean difference, *SEM* = Standard error of mean, *95% CI* = 95% confidence interval.

### Educators

Figure 3 data-driven method showed the two eye movement patterns discovered from clustering educators’ HMMs, referred to as explorative and focused eye movement patterns. The educators who exhibited an explorative eye movement pattern (n = 20) focused on the circum-oral region, whereas the focused group (n = 14) fixated on the teeth region (Fig. 3- 1 and 2). Those who adopted the explorative pattern had a 58% probability of starting by looking in the circum-oral area of the diastema (ROI1) photo, 37% starting in the no diastema circum-oral region (ROI2), and 5% starting in the nose region (ROI3). However, fixations classified as in ROI3 were all from one participant, who may not have focused on the task. Educators’ explorative pattern was also different from children’s explorative pattern. It did not have children’s ROI1 that covered both images with its centre located between the two pictures.

Educators using the focused pattern had a 48% probability of starting from viewing a broad area covering both images (ROI1), 33% probability starting from the circum-oral region of no diastema (ROI2), and 20% probability starting from the diastema (ROI3). A two-way ANOVA revealed that the two eye moment patterns were statistically significantly different (*P* < 0.05). The T-test between two eye movement groups revealed statistical significance in overall entropies, with the focused patterns having a higher entropy (*P* < 0.05). There was no statistically significant correlation between the E–F scale and overall entropy (Pearson R = 0.19, *p* = 0.23).

We discovered similar explorative and focused patterns to children’s data using the fixed ROI approach. For example, educators who adopted the explorative pattern (n = 22) had a 44% probability of starting in the image with no diastema (ROI1). Afterwards, they had a 45% probability of transitioning to the image with diastema (ROI2) and 55% to stay in the picture with no diastema (ROI1) (Fig. 3-3). They also had a 56% probability of starting in the image with diastema (ROI2); afterwards, they remained in the same image (56%) or transitioned to the image with no diastema (ROI1).

Conversely, educators who exhibited a focused eye movement pattern (n = 12) had a 58% probability of starting in the image without diastema (ROI1). Afterwards, they had a 78% probability of staying in the same image (ROI1) and only a 22% probability of moving to the photo with diastema (ROI2). They also had a 42% probability of beginning in the picture with diastema (ROI2); afterwards, they had a 33% probability of remaining in the same image (ROI2) and 67% probability of transitioning to the photo with no diastema (ROI1). The two eye movement patterns were statistically significant (*p* < 0.05). As compared with children’s focused pattern, educators’ focused pattern had a lower probability of staying in the same image (78% to go from ROI1 to ROI1 in education vs. 83% in children, and 67% to go from ROI2 to ROI2 vs. 75% in children), suggesting that they, in general, switched between two images more often than children.

The overall entropy between the two eye movement groups was significantly different [*t*(31) = 5.08, *p* = 0.02], with the focused group exhibiting a higher overall entropy. Educators adopting the focused eye movement pattern had a higher preference for the no diastema (mean = 0.64) than the educators adopting the explorative eye movement pattern (mean = 0.47, SEM = 0.06) [*t*(31) = 2.96, *p* = 0.01]. The gaze preference was compared to a hypothetical mean of 50% by a one-sample t-test. Educators in the focused group had a significantly higher gaze preference for the image without a diastema than those with diastema [*t*(10) = 2.30, *p* = 0.04; Fig. 3-4 ROI 1]. Conversely, educators in the explorative group did not have a significant bias for either image [*t*(21) = 1.08, *p* = 0.29].

The increase in mean pupil diameter is shown in Table 2 where educators following an explorative (mean = 15.76%) and focused eye movement pattern (mean = 11.65%) pupil diameter had no significant difference [*t*(28.46) = 0.88, *p* = 0.36].

## Discussion

This study is the first to use EMHMM approach with the fixed ROI technique to explore and provide objective evidence on preschool children’s attention to midline diastema. The study findings demonstrate that only children following the explorative pattern exhibited higher visual attention (eye-gaze) for the image with a diastema [mean 0.47; *t*(116) = 2.24, *p* = 0.02], while children using a focused eye movement pattern did not. Therefore, no significant differences were evident in children’s visual attention to images with and without a diastema in patterns 1 and 2. Hence, the findings did not support our hypothesis. Nevertheless, it provided valuable insights into preschool children’s eye movement patterns and visual attention.

In the data-driven method, children looked at a broad region covering both the diastema and without diastema images (ROI1 in Fig. 2 1and 2) before looking at either the circumoral regions (explorative pattern) or the teeth (focused pattern). Once they looked at these regions of either image, they switched between similar areas of the two photos during the viewing period. Children that fixated in the teeth regions managed to fixate in the ROI they started in, whether it was the ROIs with diastema or without diastema (53 and 52% for the diastema and no diastema, respectively) before they switched to the same region in the other image (25 and 29%). However, educators in the focused eye movement pattern group (20%) who first fixated on the diastema had a lower probability of looking at the diastema than children (45%). Also, 48% switched to a similar region in the no diastema image (Fig. 4). In addition, children in the 4.6–5.5y age group exhibited lower entropy than the 3.6–4.5y age group indicating higher eye movement consistency^9^. The finding confirms that younger children have less well-developed planning ability and are less experienced, and thus tend to have lower eye movement consistency behaviour.

Through the fixed ROI method, children started fixating on either diastema or without diastema pictures almost equally, meaning there was no difference in which image first caught their attention. However, children using the explorative pattern had a significantly lower gaze preference for the without diastema image (or, in other words, higher gaze preference for the with-diastema image) than those in the focused pattern group. This finding was consistent with the educators. Nevertheless, children and educators adopting the explorative pattern demonstrated different gaze preferences over the images with and without diastema. Children adopting the explorative pattern looked at the image with diastema more often than the image without diastema. In contrast, educators assuming the explorative pattern did not significantly prefer either image.

Children and educators adopting the focused eye movement pattern also showed different gaze preferences over the images with and without diastema. Children adopting the focused pattern did not have a significant preference to look at the images with or without diastema, whereas educators adopting the focused pattern had a considerable gaze preference over the image without diastema than that with diastema. In addition, the transition matrix of the HMMs revealed that educators using the focused eye movement pattern demonstrated an exciting behaviour. Once they looked at the diastema, they had a higher probability of switching to the other image than when they looked at the no diastema image in both data-driven or fixed ROI analysis methods (Fig. 3- 2 and 4). This behaviour indicates that they were biased not to continue looking at the diastema image; instead, they switched to the no diastema image. The finding may preclude that educators in the focused group shy away from the diastema.

Conversely, educators adopting the explorative pattern had no preference, demonstrated by the almost symmetric switching probability between ROI1 and ROI2 in the fixed ROI methods (Fig. 3-3). Interestingly, children in the explorative pattern using the fixed ROI method behave similarly to the transition matrix (Fig. 2-3). Thus, in children, despite having found explorative and focused groups, there is not a strong bias as educators in the transition matrix (Fig. 2).

Among children, females showed a preferential fixation that was statistically significant for the image without diastema. One explanation would be that girls noticed or fixated on the image without diastema, which did not capture their attention in boys. Children in different age groups or those who could complete the exercises did not differ in their preference or eye movement pattern groups between images with or without a diastema.

A recent trend illustrates an increased parental demand for frenotomies for either feeding difficulties or improving the children’s aesthetics to avoid negative consequences such as being bullied^19,20^. According to Medicare data, there has been a 420% increase in frenotomy rates in Australia^21^. Although this data may reflect parental awareness and demands for functional or aesthetic needs, most of these discussions do not involve the children’s desires. Therefore, significant gaps exist in our understanding of children’s perception of such dental variations. The findings of this study confirm that most children do not notice a difference in midline diastema. Consequently, this information will be valuable in confirming or refuting the parental aesthetic demands for their children and, more importantly, preventing or limiting the potential negative consequences for the child.

The participants were not asked for their opinion of the midline diastema, which can be considered a limitation. Although we could have used a qualitative evaluation, the purpose of eliminating any questions was to gauge the subject’s gaze without any bias of being told what to look at. Furthermore, children’s responses may not truly reflect their preferences in this young age group. Most surveys and interviews in children involve a proxy that may not accurately represent the child. Although fixation may not be a true reflection of choice, the gaze bias theory states gaze is actively involved in preference formation and attractiveness. A preference for objects is intrinsically connected in a positive feedback loop leading to the conscious choice^22^. One psychological measure of anticipation is the increase in pupil dilation^23^. No significant changes were evident between the preschool children or their educators in the current findings, regardless of their gender and eye movement patterns. Therefore, the diastema shown in this study did not cause arousal and may elude to despite noticing the diastema, children or educators were surprised to see one. Nonetheless, this finding may vary if the children viewed the photo of a diastema in a new setting instead of an accustomed environment like their daycare centre, which requires further investigation.

The sample size for educators is small compared to children; however, given that most centres had a ratio of educators to children as 1:5, this sample represents the typical number of educators. The difference in perspective between adults and children is shown in the ROI transition information through data-driven and fixed ROI approaches. However, the areas of interest were generally similar in the data-driven method. The only difference would be that most children started looking at the area between images. Conversely, educators fixated (excluding one outlier in Fig. 3-1a, data-driven method, ROI 3) within smaller ROIs. The nature of the study allowing children to be in their familiar environment for the eye-tracking exercise hopefully mimics what they would notice in their average day at the daycare centre, whether they would see one of their peer’s oral regions.

EMHMM has adequate spatial and temporal precision to separate discrete attentional bias measures, reflecting an individual’s intuitive and deliberate aspects. Therefore, we presented the stimuli simultaneously to understand better which of the two concurrently presented images grabs attention more readily. Also, previous studies have used similar visual tasks of showing images together, such as investigating children’s attention to healthy and unhealthy food cues^24^ and attention to attractive faces^25^. All participants viewed the pictures as illustrated in Fig. 1. This presentation overcame the centrality preference where people prefer things located in the middle of the screen compared to those at the extreme ends, i.e. left or right^26^. Also, previous research^27^ demonstrated that participants preferred items on the leftmost side, which is why we positioned the image with diastema on the right hemifield in the present study. Nevertheless, EMHHM overcomes these potential position errors as illustrated in both the data-driven and fixed ROI methods, demonstrating that all participants fixated on both pictures almost equally. Since this is the first study investigating children’s attention to midline diastema, replications and other methodological approaches such as showing the images in sequential order and using traditional eye-tracking analysis are highly encouraged.

The conventional method for analysing eye movement data has been through predefined areas/ROIs on the stimuli^28,29^ or heat maps/salience maps^30^. The limitations of these methods include that they do not adequately reflect individual differences in either spatial (such as ROI choices) or temporal dimensions (such as gaze transition among the ROIs) of eye movements. Figure 4 illustrates the heat maps generated for the present study, where red areas indicate regions participants fixated the most. The visualisation with different colours is evident qualitatively; however, there isn’t a pattern or prediction of eye movement data on a deeper level. Also, the differences between participants in the temporal dimension are not considered. For example, according to the heat maps, both children and educators have the same regions in red but cannot uncover the temporal pattern between the two images. Therefore, we conducted an in-depth individual analysis using the data-driven and fixed ROI EMHMM methods to overcome this limitation. Thus, this method is particularly suitable for examining individual differences in eye movement patterns and their associations with other cognitive measures. Also, since HMM is a probabilistic time-series model, it works well with the limited amount of data, which contrasts to deep learning methods that require large amounts of data to train effectively.

## Conclusion

Children and their educators exhibited two specific eye movement patterns: explorative and focused types. In addition, children and their educators had a difference in preference looking behaviour. Using the explorative pattern, children looked at the midline diastema more often while their educators focused on the image without the diastema. HMMs are a valuable tool in analysing eye-movement patterns in preschool children and adults.

## Supplementary Information

Supplementary Information 1.

Supplementary Information 2.

Supplementary Information 3.

## Data Availability

The datasets generated during the current study are available from: https://osf.io/936mw/?view_only=d6ed1db7f866423bb667ebc22d953b69.
