# Supplementary material for: Eye movement analysis of children’s attention for midline diastema
Source: Sci Rep. 2022 May 6;12:7462. doi: 10.1038/s41598-022-11174-z (PMC9076614; doi:10.1038/s41598-022-11174-z)
Supplement: Supplementary file 1 — Supplementary Information 1. [file 41598_2022_11174_MOESM1_ESM.docx]

Appendix

**Title: Eye movement analysis of children’s attention for midline diastema**

Authors: *Vanessa Cho, Janet Hsiao, Antoni Chan, Hien Ngo, Nigel King, Robert Anthonappa*

Material and methods:

*Data analysis*

This method incorporates individual differences in eye movement spatial(eye fixation locations) and temporal dimensions(the order of eye fixation locations) to provide a quantitative measure of individual differences in the eye movement pattern ^[1]^. Furthermore, combining EMHMM with the data mining technique, co-clustering facilitates identifying participant groups with consistent eye-movement patterns while viewing clinical images with varying layouts. Thus, this innovative approach is suitable for exploring individual differences in eye movement patterns and their associations with the participant's cognitive measures ^[2]^.

Using EMHMM, each participant's eye movements were summarised using a hidden Markov model (HMM), with its hidden states corresponded to the viewed regions of interest (ROIs). A hidden state sequence generated a sequence of the viewed ROIs under a Markov process. Person-specific ROIs were identified based on the eye fixation distributions. The transitions among the ROIs were summarised into a transition matrix showing the probability of eye gaze moving from a previously viewed ROI to the current ROI. Subsequently, individual HMMs were clustered into groups according to the similarities of their ROIs and ROI sequences to discover representative eye movement patterns among the participants.

Following previous studies ^[1,2,3]^, we clustered individual HMMs into two representative patterns: Pattern 1 and Pattern 2. The similarity of a participant's eye movement data to a suggestive pattern could be quantified using the log-likelihood of the participant's eye movement data being generated by the representative model. We then assessed each participant's eye movement pattern using a 1-2 scale, defined as (L1 – L2)/(|L1|+|L2|), where L1 and L2 stand for the log-likelihoods of the participant's eye movement data being generated by Pattern 1 and Pattern 2 respectively ^[2,4,5,6]^. Eye movement consistency was assessed using the HMM's overall entropy, where entropy was a measure of regularity or predictability of eye movements ^[5]^. For example, a higher entropy indicated more randomness or variability within an individual's eye movements. Raw data were analysed using EMHMM ^[1]^ and GraphPad Instat (California, USA).

References:

1. Chuk T, Chan AB, Hsiao JH. Understanding eye movements in face recognition using hidden Markov models. *J. Vis.* **14**(11):8. (2014).
2. Hsiao JH, Lan H, Zheng Y, Chan AB. Eye Movement analysis with Hidden Markov Models (EMHMM) with co-clustering. *Behav. Res. Methods.* **30**:1-4. (2021)
3. Chuk T, Chan AB, Shimojo S, Hsiao JH. Eye movement analysis with switching hidden Markov models. *Behav. Res. Methods.* **52**:1026-1043. (2020)
4. An J, Hsiao JH. Modulation of mood on eye movement pattern and performance in face recognition. *Emotion.* **21**:617-630. (2021)
5. Chan CY, Chan AB, Lee TM, Hsiao, JH. 2018. Eye-movement patterns in face recognition are associated with cognitive decline in older adults. *Psychon. Bull. Rev.* **25**(6):2200-2207. (2018)
6. Zhang J, Chan AB, Lau EYY, Hsiao JH. 2019. Individuals with insomnia misrecognize angry faces as fearful faces while missing the eyes: an eye-tracking study. *Sleep.* **42**(2):1-11. (2019)


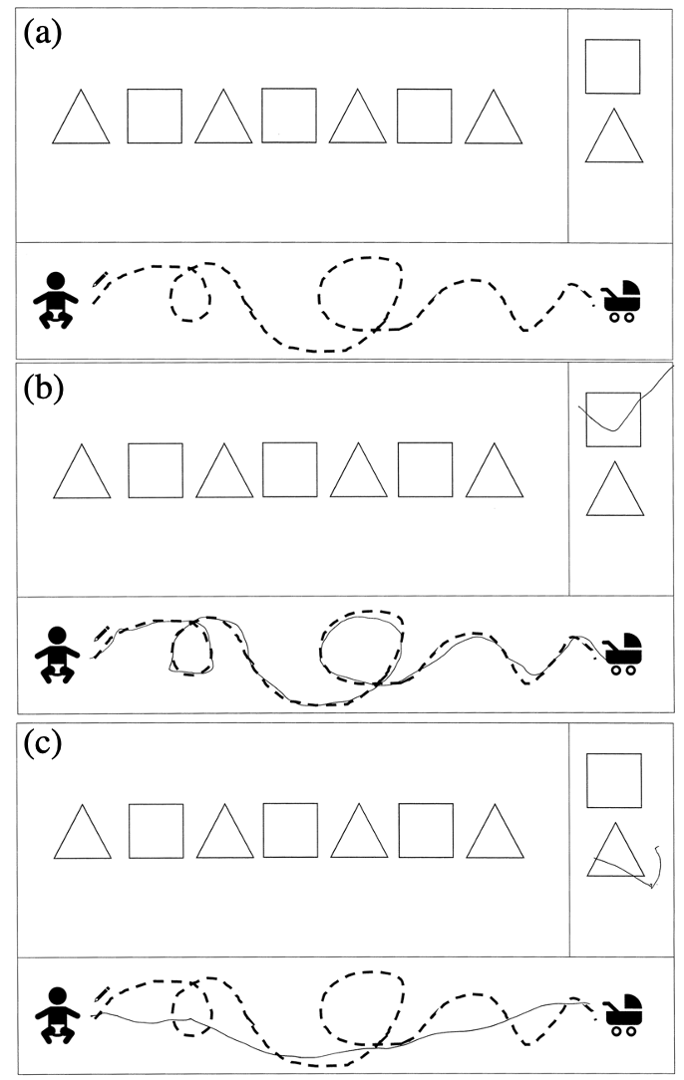


**Appendix Figure 1.** (a) Before viewing the pictures, all children were presented with two activities: complete the pattern and join the dots in a line (b) all exercises complete or (c) incomplete
