# Supplementary material for: Eye movement analysis of children’s attention for midline diastema
Source: Sci Rep. 2022 May 6;12:7462. doi: 10.1038/s41598-022-11174-z (PMC9076614; doi:10.1038/s41598-022-11174-z)

Appendix Figure 2.

Changes in average pupil diameter (mm) over time (10 seconds) between (a) distributed and selective eye movement pattern groups, (b) gender and (c) age groups.


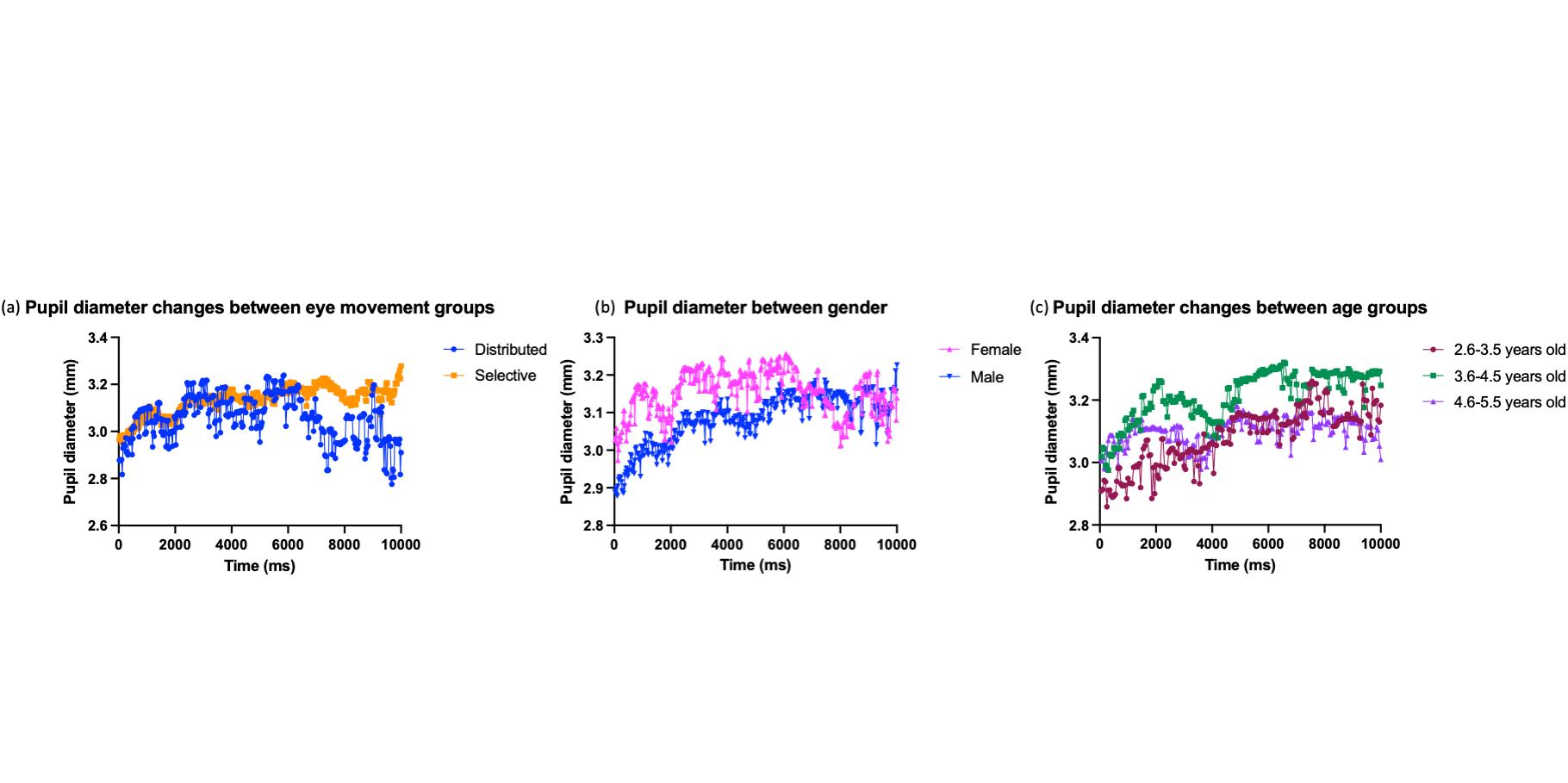


Appendix Figure 3.

Changes in average pupil diameter (mm) over time (10 seconds) between areas of interest (diastema or no diastema) for variables for

1. children, (b) selective eye movement pattern group, (c) distributed eye movement pattern group, (d) age, (e) gender and (f) educators


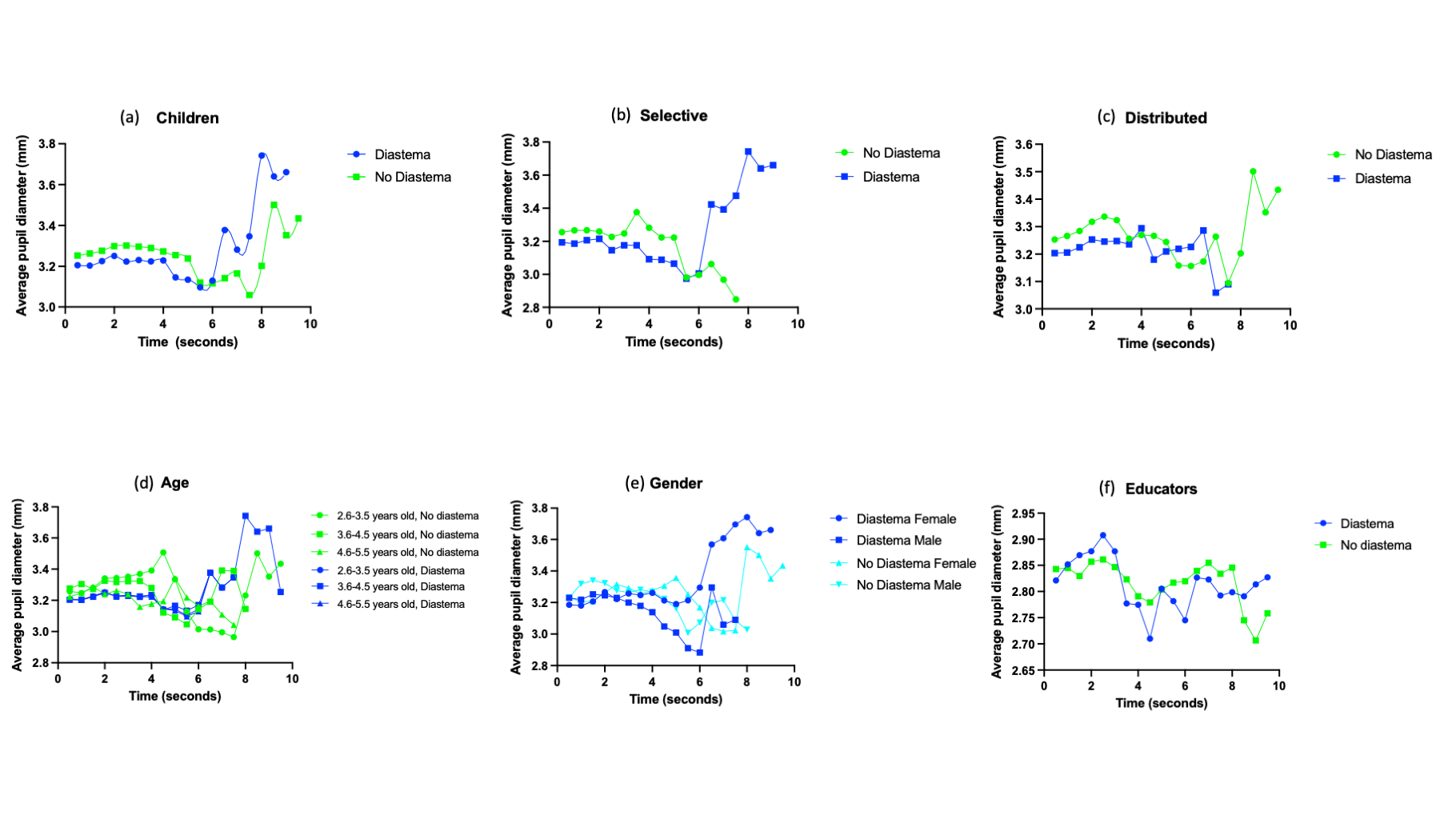

Supplement: Supplementary file 2 — Supplementary Information 2. [file 41598_2022_11174_MOESM2_ESM.docx]
