# Supplementary material for: Eye movement analysis of children’s attention for midline diastema
Source: Sci Rep. 2022 May 6;12:7462. doi: 10.1038/s41598-022-11174-z (PMC9076614; doi:10.1038/s41598-022-11174-z)
Supplement: Supplementary file 3 — Supplementary Information 3. [file 41598_2022_11174_MOESM3_ESM.docx]

| **Gender** | **Ethnicity** | **Age (years)** | | | **Total** |
| --- | --- | --- | --- | --- | --- |
|  |  | 2.6-3.5 | 3.6-4.5 | 4.6-5.5 |  |
| Male | White | 25 | 19 | 10 | 54 |
| n=83 | Asian | 3 | 9 | 7 | 19 |
|  | Black | 2 | 2 | 6 | 10 |
| Female | White | 18 | 13 | 14 | 45 |
| n=72 | Asian | 6 | 7 | 5 | 18 |
|  | Black | 0 | 2 | 7 | 9 |
| **Total** |  | 54 | 52 | 49 | 155 |

**Appendix Table 1.** Study participants' details based on ethnicity, gender, age and activity completion. Note: all children were born in Australia.
